# Injury-induced interleukin-1 alpha promotes Lgr5 hair follicle stem cells de novo regeneration and proliferation via regulating regenerative microenvironment in mice

**DOI:** 10.1186/s41232-023-00265-7

**Published:** 2023-02-20

**Authors:** Guang Yang, Haiyan Chen, Qun Chen, Jiayi Qiu, Mulan Qahar, Zhimeng Fan, Weiwei Chu, Edward E. Tredget, Yaojiong Wu

**Affiliations:** 1 State Key Laboratory of Chemical Oncogenomics, and the Institute of Biopharmaceutical and Health Engineering (iBHE), Shenzhen International Graduate School, Tsinghua University, Shenzhen, 518055 China; 2 Department of Burn and Plastic Surgery, Shenzhen Institute of Translational Medicine, Shenzhen Second People’s Hospital, The First Affiliated Hospital of Shenzhen University, Shenzhen, 518035 China; 3 Division of Nephrology, Peking University Shenzhen Hospital, Shenzhen, 518036 China; 4 Tsinghua-Berkeley Shenzhen Institute (TBSI), Tsinghua University, Shenzhen, 518055 China; 5 Faculté Des Lettres, Sorbonne Université (Paris Sorbonne, 75006 Paris IV), Paris, France; 6 Department of Surgery, Division of Critical Care, University of Alberta Hospital, Edmonton, AB ABT6G2B7 Canada

**Keywords:** Hair follicles, Lgr5 stem cells, Interleukin-1alpha, Wound healing, Regeneration, HaCaT, SKPs

## Abstract

**Background:**

The hair follicles (HFs) are barely regenerated after loss in injuries in mammals as well as in human beings. Recent studies have shown that the regenerative ability of HFs is age-related; however, the relationship between this phenomenon and the stem cell niche remains unclear. This study aimed to find a key secretory protein that promotes the HFs regeneration in the regenerative microenvironment.

**Methods:**

To explore why age affects HFs de novo regeneration, we established an age-dependent HFs regeneration model in leucine-rich repeat G protein-coupled receptor 5 (Lgr5) + /mTmG mice. Proteins in tissue fluids were analyzed by high-throughput sequencing. The role and mechanism of candidate proteins in HFs de novo regeneration and hair follicle stem cells (HFSCs) activation were investigated through in vivo experiments. The effects of candidate proteins on skin cell populations were investigated by cellular experiments.

**Results:**

Mice under 3-week-old (3W) could regenerate HFs and Lgr5 HFSCs, which were highly correlated with the immune cells, cytokines, IL-17 signaling pathway, and IL-1α level in the regeneration microenvironment. Additionally, IL-1α injection induced de novo regeneration of HFs and Lgr5 HFSCs in 3W mouse model with a 5 mm wound, as well as promoted activation and proliferation of Lgr5 HFSCs in 7-week-old (7W) mice without wound. Dexamethasone and TEMPOL inhibited the effects of IL-1α. Moreover, IL-1α increased skin thickness and promoted the proliferation of human epidermal keratinocyte line (HaCaT) and skin-derived precursors (SKPs) in vivo and in vitro, respectively.

**Conclusions:**

In conclusion, injury-induced IL-1α promotes HFs regeneration by modulating inflammatory cells and oxidative stress-induced Lgr5 HFSCs regeneration as well as promoting skin cell populations proliferation. This study uncovers the underlying molecular mechanisms enabling HFs de novo regeneration in an age-dependent model.

## Introduction

Adult skin consists of a keratinized stratified epidermis and an underlying layer of dermis. The hair follicle (HF), sebaceous gland and sweat gland, namely the appendage of the skin are derived from a single layer of multipotent progenitors during skin morphogenesis [1]. HFs are important cutaneous appendages that maintain skin function and self-renewal [2]. In postpartum humans, however, deep injuries to the skin heal with scar formation but do not regenerate, and the epidermal appendages lost at the injury site do not regenerate [3]. Therefore, HFs regeneration helps to restore the normal function of the patient’s skin. It has been a challenge to regenerate the HFs.

Recently, Ito et al. observed that large wounds could induce HFs neogenesis via activating the WNT-mediated pathway in adult mice [4]. For example, a 1 cm^2^ wound was sufficient to induce HFs neogenesis in 3-week-old (3W) mice, whereas older mice required larger wounds (2.5 cm^2^) to trigger HFs neogenesis. Interestingly, this study not only demonstrates that HFs de novo regeneration correlates with the WNT pathway, but also with age and wound size. Later, several studies have found a variety of factors that can regulate HFs regeneration by mediating the stem cell niche. *Hoxc* genes promote regional HFs regeneration via inducing reprogram mesenchymal dermal papilla (DP) cells and altering the epithelial stem cells’ regenerative potential [5]. IL-1α promotes hair follicle stem cells (HFSCs) and interfollicular epidermal stem cells proliferation through mediating dermal fibroblasts and activating gamma delta T cells (γδT-cells) [6]. During wound healing, sonic hedgehog overexpression promotes HFs neogenesis by reinstalling a regenerative dermal niche [7]. Injured HFs release CCL2 leads to M1 macrophage accumulation and tumor necrosis factor-alpha (TNF-α) secretion, driving HFSCs regeneration [8]. These studies suggest that HFs regeneration after injury is feasible, but it requires specific conditions to drive it.

The key to tissue regeneration induced by the regenerative microenvironment is the activation of stem cells, which are characterized by their extraordinary ability to self-renew through cell division, and differentiate into a wide range of tissue-specific cells in response to endogenous or external stimuli. Because of these regenerative effects, stem cells in their specific niches repair tissue after injury/disease and maintain tissue throughout life. Generally, stem cell stemness and tissue repair ability decrease with age [4, 9–12]. The reduced regenerative capacity is considered as an expected response, in which wound healing takes precedence over functional recovery, resulting in scar tissue formation [13]. Deviation from conventional repair may lead to some unpredictable pathological situations, which are often observed in old groups [14]. Similarly, the regenerative capacity of HFs decreases with age in mice [4]. HFs can be de novo regenerated in the wound center. This indicates that, compared with older mice, there are special factors in the wound regeneration microenvironment in younger mice that can drive HFs regeneration.

Leucine-rich repeat-containing G protein-coupled receptor 5 (Lgr5), a receptor involved in the WNT signaling pathway, is a bona fide marker of stem cells in various epithelial tissues including the HFs and the intestine [15–17]. In the gut, Lgr5 cells are not necessary for gut homeostasis [18], but they are essential for intestinal regeneration after radiation [19]. Similarly, Lgr5 HFSCs are necessary for hair regeneration and wound healing but not for homeostasis [2, 20]. Depletion of Lgr5 HFSCs inhibits hair regeneration; however, this phenomenon is reversible [21]. After depletion of Lgr5 HFSCs, CD34 HFSCs can transdifferentiate into Lgr5 HFSCs and regenerate HFs again. However, the role of Lgr5 HFSCs in wound healing and the molecular mechanisms that regulate stem cell plasticity remain unclear.

In our experience, HFSCs regeneration can be divided into two stages after wound, the first stage is de novo regeneration and the second stage is activation and proliferation. As mentioned earlier, HFSCs de novo regeneration is driven by special conditions. However, the presence of HFSCs does not mean that the HFSCs are activated, in other words, that the HFSCs do not become a complete follicular unit or that the HFs are not fully regenerated. Role of HFSCs in wound healing is very specific. Generally, HFSCs will be housed in a relatively stable structure that is not involved in other tissue activities. After injury, HFSCs migrate to the wound surface and promote epithelialization [22–25]. It is generally accepted that the reprogrammed HFSCs will not re-dedifferentiate/transdifferentiate into HFSCs. In patients, skin healing is usually replaced by scar healing. However, in patients who have been discharged for many years, we have occasionally observed a small number of HFs in the center of the regenerated skin. These studies and observations all point in one direction, that the activation of HFSCs requires a specific condition or a long period of time. The wounds may have regenerated HFSCs, but not transformed into a complete follicular unit. Moreover, activation and proliferation of HFSCs are closely related to the HFs cycle, which includes anagen, catagen, and telogen [17, 26]. Activated HFSCs promote cell proliferation and enable HFs to enter the anagen phase. Conversely, HFSCs stop growing, HFs shrink, and hair stops growing or shedding. In our experience, HFs in C57BL/6 mice transition from anagen to telogen at 2–4 weeks and 6–8 weeks of age. A change of skin from white to blue-black can be observed at 10–14 days after dorsal epilation, signifying HFs regeneration. After 21 days, the new hair will be as long as the original hair.

We hypothesized that there are special factors in the regeneration microenvironment of younger mice that can induce HFs and HFSCs de novo regeneration after wounding. This study aimed to explore a key secretory protein that drives HFs and HFSCs de novo regeneration and investigate the key mechanisms involved. For this purpose, we established an age-dependent wound healing mice model to observe the effect of age on HFs regeneration. The differences between the regenerative microenvironments were explored by protein array and quantitative reverse transcription PCR (qRT-PCR), and key proteins were screened. To investigate the effect of candidate proteins on Lgr5 HFSCs and to elucidate the importance of Lgr5 HFSCs for HFs regeneration, we generated Lgr5 + /mTmG transgenic mice that displayed green fluorescence in HFSCs and red fluorescence in the cell membrane. In addition, the mechanism of the candidate proteins was verified by cell experiments and intervention experiments. This study has important implications for the regeneration of skin appendages after wounding.

## Methods

### Animals

All animal experiments were performed with the approval of the Institutional Animal Care and Use Committee at Tsinghua University. The current protocols were also approved by Bioethics Committee of Tsinghua University Shenzhen International Graduate School. Lgr5 + /mTmG mice (C57BL/6 background) were housed in a laminar flow cabinet and maintained on normal diets (1025, HFK, Beijing, China) in Shenzhen Center for Disease Control and Prevention (SCDCP). Mice were anaesthetized with isoflurane (970–00,026-00, RWD, Shenzhen, China) and sacrificed by CO_2_. One allele of Lgr5 is linked to a green fluorescent protein (GFP) that fluoresces green, while mTmG fluoresces red at the cell membrane.

### Models

All experiments were performed on littermates. (1) To investigate the skin regeneration ability in mice, a round wound was made by a biopsy punch without any treatment. (2) To investigate the role of IL-1α in HFs de novo regeneration, 1 μg IL-1α (Z02912, GenScript, Nanjing, China) was infused in 5 mm diameter wound of 3W mice. The wound was covered with a Tegaderm film (1624W, 3 M, MN, USA), and then removed 3 days later. (3) To investigate the role of inflammation and oxidative stress in HFs regeneration, mice were treated with dexamethasone (Dex) cream (Teyi, Guangdong, China. Anti-inflammatory synthetic glucocorticoid) to the back for 7 days or treated with TEMPOL [3 mmol/L water. GS16245, Cool Chemistry, Beijing, China. Reactive oxygen species (ROS) scavenger.] for 21 days. (4) To investigate the role of IL-1α in adult mice, 3 μg IL-1α was injected subcutaneously in adult mice. Smear dexamethasone cream was applied to the back for 7 days. Mice were sacrificed after 14 days. IL-1α was dissolved in phosphate-buffered saline (PBS) with 0.1% bovine serum albumin (BSA), and then mix with Matrigel (356,230, BD, New York, USA). PBS: Matrigel = 5 μl: 50 μl.

### BCIP/NBT staining (alkaline phosphatase, AP)

Dorsal skin samples were collected and stained with BCIP/NBT kit (C3206, Beyotime, Beijing, China) overnight. Photos were taken by microscope (MZ95, Leica, Weztlar, Germany).

### Protein array

Dorsal skin tissues (2 mm around the wound) were collected and preserved in PBS at 4 ℃. Tissues were ruptured by scissors, and tissue fluid samples were collected by centrifuge and sent to Raybiotech (Guangzhou, China) for protein array (GSM-CAA-4000, Raybiotech, Atlanta, USA). Results were standardized and quantified.

### qRT-PCR

Total RNA was isolated from mouse dorsal skin using TRIzol® reagent (9109, Takara, Kyoto, Japan). The cDNA was synthesized using PrimeScript RT reagent Kit (RR047A, Takara, Kyoto, Japan). The qRT-PCR was performed with ABI-7300 (ABI, Foster, CA, USA) using SYBR Green (B21203, Bimake, Shanghai, China) according to the manufacturer’s instructions [27]. Macrophage subtype biomarkers refer to the previous literature for selection [28]. Primers see Table 1. *Gapdh* was selected for reference.

**Table 1:** Primers

Full name	Gene ID	Short name	Forward primer	Reverse primer
Interleukin 1 beta	16,176	*Il1b*	5'-AAGAGCTTCAGGCAGGCAGTATCA-3'	5'-TGCAGCTGTCTAATGGGAACGTCA-3'
Interleukin 6	16,193	*Il6*	5'-TCTATACCACTTCACAAGTCGGA-3'	5'-GAATTGCCATTGCACAACTCTTTC-3'
Chemokine (C–C motif) ligand 2	20,296	*Ccl2*	5'-CCAGCCTACTCATTGGGATCA-3'	5'-CTTCTGGGCCTGCTGTTCA-3'
Tumor necrosis factor	21,926	*Tnf*	5'-ACGTCGTAGCAAACCACCAA-3'	5'-GCAGCCTTGTCCCTTGAAGA-3'
Chitinase-like 3	12,655	*Chil3*	5'-ACTTTGATGGCCTCAACCTGGACT-3'	5'-TGGAAGTGAGTAGCAGCCTTGGAA-3'
Resistin like alpha	57,262	*Retnla*	5'-ACTGCCTGTGCTTACTCGTTGACT-3'	5'-AAAGCTGGGTTCTCCACCTCTTCA-3'
Arginase	11,846	*Arg1*	5'-ACCTGGCCTTTGTTGATGTCCCTA-3'	5'-AGAGATGCTTCCAACTGCCAGACT-3'
Interleukin 1 alpha	16,175	*Il1a*	5'-TCTATGATGCAAGCTATGGCTCA-3'	5'-CGGCTCTCCTTGAAGGTGA-3'
Macrophage galactose N-acetyl-galactosamine-specific lectin 2	216,864	*Mgl2*	5'-TTAGCCAATGTGCTTAGCTGG-3'	5'-GGCCTCCAATTCTTGAAACCT-3'
Mannose receptor, C type 1	17,533	*Mrc1*	5'-GTGCTGGTTGTGATAGCCATC-3'	5'-TGCTGACACTTACCATCAGGT-3'
Signaling lymphocytic activation molecule family member 1	27,218	*Slamf1*	5'-CAGAAATCAGGGCCTCAAGAG-3'	5'-CACTGGCATAAACTGTGGTGG-3'
Interleukin 12b	16,160	*Il12b*	5'-TGGTTTGCCATCGTTTTGCTG-3'	5'-ACAGGTGAGGTTCACTGTTTCT-3'
Interleukin 1 receptor, type I	16,177	*Il1r1*	5'-GTGCTACTGGGGCTCATTTGT-3'	5'-GGAGTAAGAGGACACTTGCGAAT-3'
Forkhead box P3	20,371	*Foxp3*	5'-ACAACCTGAGCCTGCCACAGT-3'	5'-GCCCACCTTTTCTTGGTTTTG-3'
Cytotoxic T-lymphocyte-associated protein 4	12,477	*Ctla4*	5'-TGGAGTCCTTCATAGTTAGG-3'	5'-GCAAGATGGTGAGTGTGATGTT-3'
Transforming growth factor, beta 1	21,803	*Tgfb1*	5'-CAACGCCATCTATGAGA-3'	5'-AAGCCCTGTATTCCGTCTCC-3'
Glyceraldehyde-3-phosphate dehydrogenase	14,433	*Gapdh*	5'-AGGTCGGTGTGAACGGATTTG-3'	5'-GGGGTCGTTGATGGCAACA-3'

### Flow cytometry

Skin samples were incubated with 400 μl PBS (0.35% dispase II, D4693, Sigma-Aldrich, USA) at 4 ℃ for 3 h. The sample was then wash, cut, and incubated with 200 μl PBS (1% collagenase, C0130, Sigma-Aldrich, USA) at 37 ℃ for 4 min and was filtered with 70-micron screen and centrifuged at 1500 g for 5 min. Wash and incubated with F4/80 antibody (123,110, Biolegend, San Diego, CA, USA) at 4 ℃ for 30 min. Wash and centrifuged at 1500 × *g* for 5 min. Filtration with 40-micron screen and measured by fluorescence-activated cell sorting (FACS, C6 Plus, BD, New York, USA).

### Histology

Dorsal skin tissues were collected and preserved in 4% PFA overnight. Paraffin (4.06.31172, Citotest, Hangzhou, China) embedded samples (8 μm) were stained by hematoxylin and eosin (H&E) solutions (PH0516, Phygene, Fuzhou, China) or Sirius Red (T1470, Tsinglight, Wuhan, China) according to the manufacturer’s protocols [29]. Skin samples of Lgr5 + /mTmG mice were embedded in optimum cutting temperature compound (OCT, #4583, Sakura, Torrance, USA) and sliced to 8-micron (CM1950, Leica, Weztlar, Germany). Photos were taken by microscope (Axiocam 503, Zeiss, Jena, Germany; X-Cite 120Q, Excelitas, Toronto, Canada).

### Cell culture

Five thousand number of HaCaT cells were incubated in 96-well-plate with DMEM (10–014-CVR, Corning, New York, USA) + 10% FBS (04–001-1A, BI, Beit Haemek, Israel) for 24 h. Then treated with IL-1α for 48 h and CCK-8 (T1210, Tsinglight, Wuhan, China) for another 2 h. The OD values were recorded for samples (Epoch, BioTek, Winooski, USA). Skin-derived precursors (SKPs) isolation and incubation protocols were described as before [30]. Ten thousand number of SKPs were incubated in 6-well plate for 72 h. Cell size and number were calculated under the microscope.

### Statistics

Data are expressed as the mean ± standard deviation (SD). Statistical analysis was performed with the unpaired two-tailed Student’s *t*-test using GraphPad Prism software (Version 6, San Diego, USA). A value of *P* ≤ 0.05 was considered as statistical significance.

## Results

### Immature mice have better HFs and HFSCs de novo regeneration ability

**Fig. 1:**
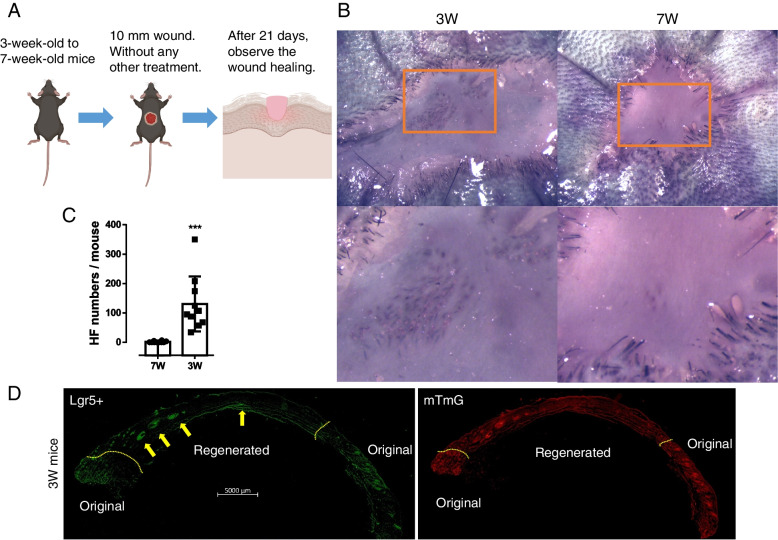
HFs de novo regeneration in mice. **A** Experimental steps. **B** Representative pictures of HFs de novo regeneration after wounding. **C** 3W mice regenerated more HFs than 7W mice (7W vs 3W, 1.5 ± 0.82 vs 130.5 ± 29.51, *n* = 10). **D** Representative photos of Lgr5 HFSCs regeneration in 3W Lgr5 + /mTmG mice. Yellow arrows point to regenerated Lgr5 HFSCs. The yellow dashed line represents the wound margin. ***P < 0.005

To investigate the effect of age on the HFs de novo regeneration, a 10-mm-diameter wound was incised on the dorsal skin of mice with different ages (Fig. 1A). After 21 days, the mice were sacrificed and the HFs regeneration in the wound center was observed (Fig. 1B, C). Regenerated HFs were clearly observed in 3W mice. This phenomenon was difficult to observe as the mice got older. 7W mice had few regenerated HFs. To further confirm the HFs de novo regeneration, we used 3W Lgr5 + /mTmG mice. As expected, green fluorescence appeared in the regenerated tissue, proving the Lgr5 HFSCs de novo regeneration (Fig. 1D). This indicates that the wound healing model for age-dependent HFs regeneration is successful. The de novo regeneration ability of HFs and Lgr5 HFSCs in mice reduced significantly with age, and it was difficult to observe the HFs de novo regeneration in mice over 3-week-old.

### Differences in regenerative microenvironment between 3 and 7W mice after wounding

**Fig. 2:**
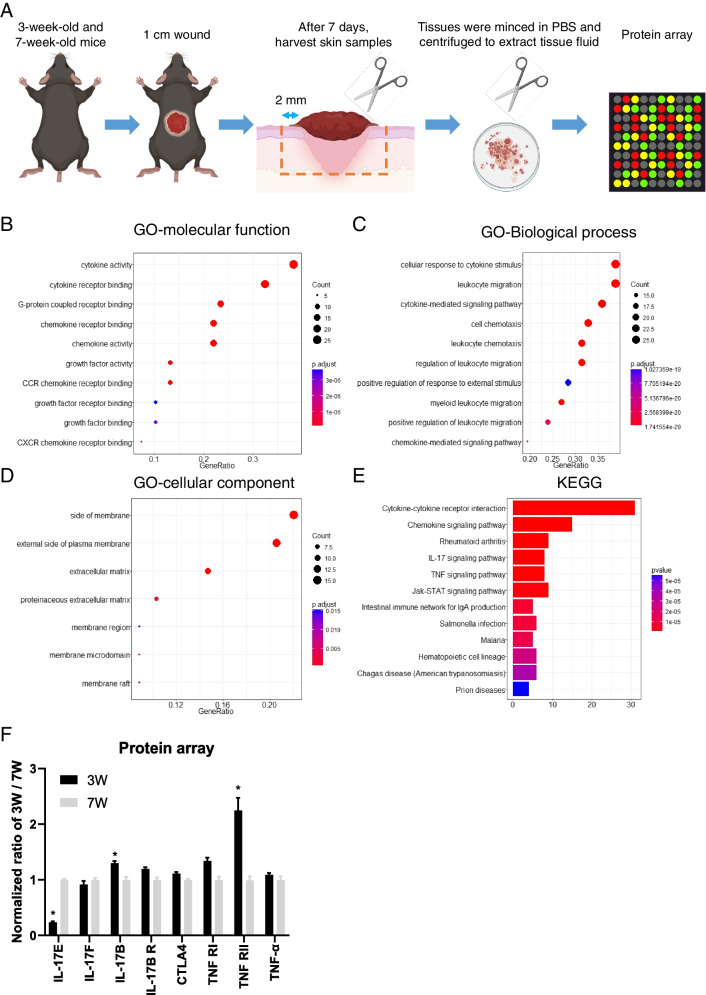
The biological differences between 3 and 7W mice in wound healing. **A** Experimental steps. **B**, **C**, **D** GO analysis of protein array in molecular function, biological process, and cellular component. **E** KEGG analysis of protein array. **F** Normalized expression levels of IL-17 family, regulatory T cells biomarker CTLA4 and TNF family. *P < 0.05

Stem cells are influenced by the regenerative microenvironment. To compare differences in exocrine proteins during wound healing between immature and young mice, 3W and 7W mice were selected because their HFs were in telogen to avoid the effects of activated HFSCs [2]. Protein array analysis showed significant differences in the composition of wound tissue fluid on the 7th day after wounding (Fig. 2A). Gene Ontology (GO) analysis of molecular functions indicated that the differential expression levels of proteins in the cytokine, chemokine, and growth factor classes may be the main cause of HF regeneration (Fig. 2B). Bioprocess analysis indicated that leukocytes, cytokines, and their related signaling pathways may contribute to skin regeneration (Fig. 2C). Analysis of cellular components showed that skin regeneration capacity was highly correlated with membrane components and extracellular matrix (Fig. 2D). To confirm these results, the Kyoto Encyclopedia of Genes and Genomes (KEGG) was used to analyze this information. The results showed significant differences between 3 and 7W mice in cytokines, chemokines and their associated signaling pathways, especially in interleukin (IL)-17 and TNF-α (Fig. 2E).

IL-17 is a pro-inflammatory cytokine produced by activated CD4( +) T cells. TNF-α protein is mainly produced by immune cells and is also a cytokine involved in inducing inflammation. Interestingly, when we analyzed the expression levels of IL-17 family (IL-17E, IL-17F, IL-17B, IL-17B R), regulatory T cells (Tregs) biomarker (CTLA4) and TNF family (TNF RI, TNF RII, and TNF-α), the trends were not consistent (Fig. 2F). This may involve alterations in numerous upstream and downstream regulatory signals, suggesting that the regeneration of skin and HFs is a complex process. Consequently, the whole process of skin regeneration is closely related to immune cells and cytokines.

### HFs regeneration is correlated with inflammatory cell phenotypes

**Fig. 3:**
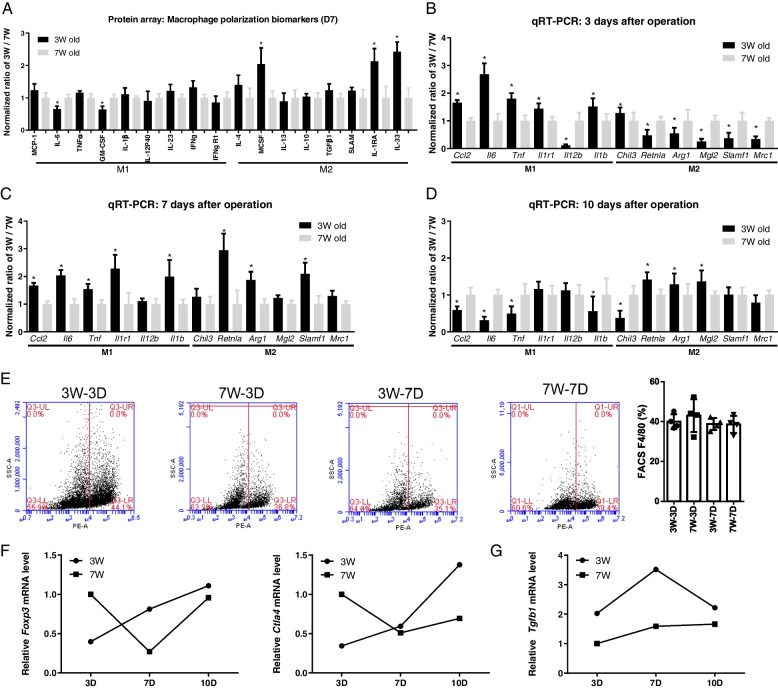
Immune cells phenotype in wound healing. **A** At day 7, 3W mice have less M1 and more M2 protein biomarkers. **B** At day 3, 3W mice have more M1 and less M2 mRNA levels (*n* = 6–8). **C** At day 7, 3W mice have more M1 and M2 mRNA levels (*n* = 6–8). **D** At day 10, 3W mice have less M1 and more M2 mRNA levels (*n* = 6–8). **E** At third day (D3) and seventh day (D7), there is no difference in macrophage infiltration between 3 and 7W mice. (3W-3D, 40.00 ± 1.864; 7W-3D, 43.00 ± 4.106; 3W-7D, 38.78 ± 1.511; 7W-7D, 38.61 ± 2.170; *n* = 4.) **F** *Foxp3* and *Ctla4* mRNA levels at 3D, 7D, and 10D, respectively. (*n* = 3) **G** 3W mice have higher *Tgfb1* mRNA expression levels (*n* = 3). *P < 0.05

Different inflammatory cell subtypes have diverse effects on tissue regeneration. For example, macrophages can be polarized to the pro-inflammatory (M1) and anti-inflammatory (M2) phenotype. Normally, prolonged M1 is harmful to the organism, and M2 is responsible for tissue repair. To understand the role of macrophage phenotype in wound healing, M1 and M2 biomarkers were analyzed. For easy understanding, 7W mouse expression level was used as a reference standard. The protein array analysis showed that 3W mice had fewer M1 and more M2 biomarkers (Fig. 3A). To understand the dynamic change of macrophage polarization, tissue samples were measured. Compared to 7W mice, 3W mice had more M1 and less M2 on day 3 after the operation (Fig. 3B). And 3W mice had more M1 and M2 on day 7 (Fig. 3C). On day 10, 3W mice had less M1 and more M2 (Fig. 3D). To quantify the number of macrophages infiltrated into the wound, skin samples were collected on day 3 and 7 and measured by flow cytometry. The results showed no marked difference between 3 and 7W mice (Fig. 3E). It suggests that HFs regeneration is associated with a higher M1/M2 ratio in the early stages of injury and a lower M1/M2 ratio later, independent of macrophage numbers.

A recent study found that Tregs contribute to HFSCs proliferation [31]. To identify the changes of Tregs in HFs wound healing, mRNA expression levels were measured by qRT-PCR. It showed that *Foxp3* and *Ctla4* (Tregs biomarkers) expression levels may gradually increase in 3W mice after wounding, and finally were higher than 7W mice on day 10 (Fig. 3F). In addition, transforming growth factor-beta 1 (*Tgfb1*) is critical for recruiting macrophages, inducing Tregs, and regulating HFs cell fate [32–34]. We measured the *Tgfb1* expression levels and found that 3W mice had higher levels of *Tgfb1* at all time points (Fig. 3G). It means that Tregs and *Tgfb1* are also involved in skin regeneration and are associated with HFs regeneration.

### HFs de novo regeneration is associated with upregulated levels of IL-1α

**Fig. 4:**
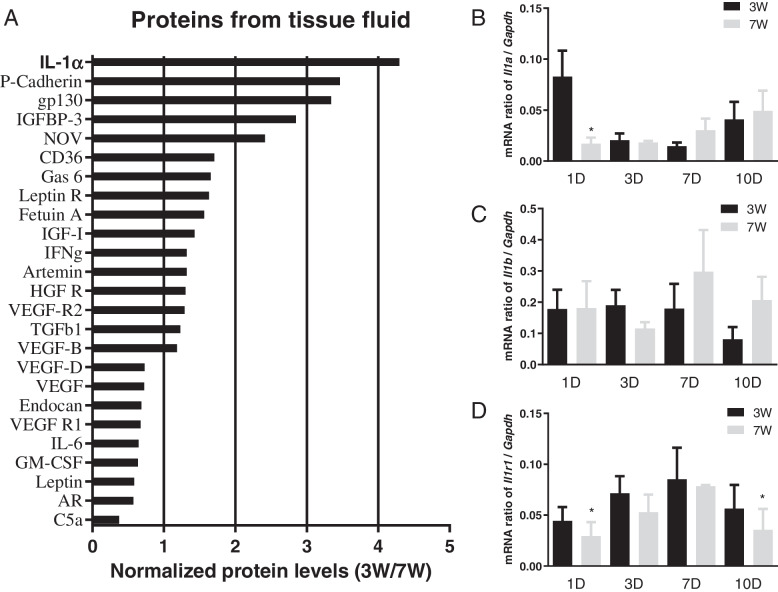
IL-1α is highly expressed in 3W mice. **A** Relative protein levels between 3 and 7W mice at seventh day after the operation. (BCD) Relative mRNA expression levels of *Il1a*, *Il1b and Il1r1* at 1st (1D), 3rd (3D), 7th (7D), and 10th (10D) day after wounding. (*n* = 6–8) *P < 0.05

Next, to screen the key proteins influencing HFs de novo regeneration, we further compared the protein expression levels from tissue fluid. The protein array analysis showed that 3W mice had significantly higher IL-1α levels in tissue fluid on day 7 post-operation (Fig. 4A). To track the dynamic expression levels of the IL-1 family during wound healing, mRNA samples were isolated from the 1st, 3rd, 7th, and 10th day post-operation. Notably, *Il1a* was highly expressed 24 h after injury in 3W mice, and there is no significant difference in other time points (Fig. 4B). *Il1b* was not significantly different between groups (Fig. 4C). *Il1r1* level in 3W mice was higher on day 1 and day 10 (Fig. 4D). This suggests that injury directly induces mRNA transcription of *Il1a*, after which IL-1α protein persists in the regenerative microenvironment for a long time.

### IL-1α accelerates de novo regeneration of HFs and Lgr5 HFSCs in 3W mice

**Fig. 5:**
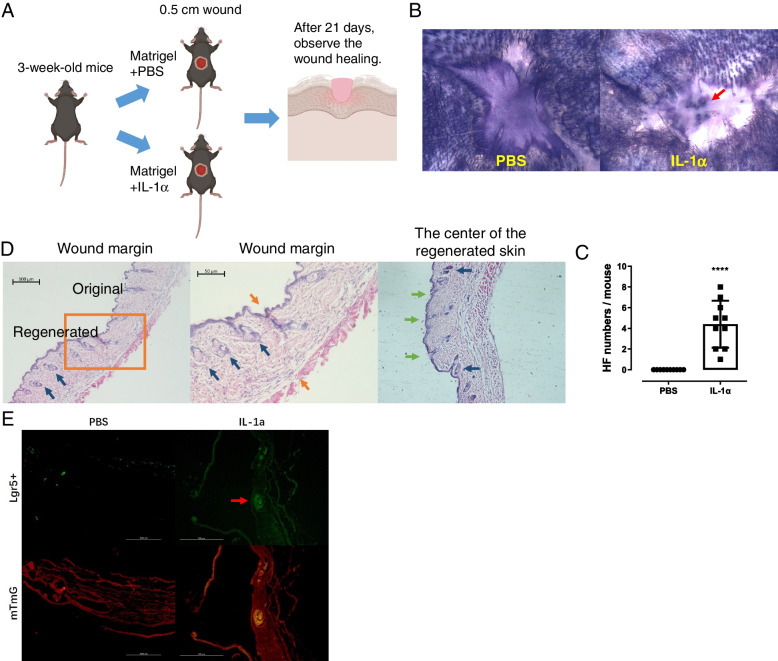
IL-1α promoted de novo HF regeneration in 3W mice with a 0.5-cm-diameter wound. **A** Experimental steps. **B** Representative pictures of HF regeneration. The red arrow points to the regenerated HF. **C** If the diameter of the wound is less than 0.5 cm, 3W mice could not regenerate HFs. But IL-1α significantly promoted the de novo HFs regeneration (PBS vs IL-1α, 0.0 ± 0.0 vs 4.400 ± 0.7180, *n* = 10). **D** Representative pictures of regenerated HFs. The orange arrows point to the boundary between regenerated and original tissue. The green arrows point to the HF have grown out from the regenerated skin. The blue arrows point to the HF were still growing in the regenerated skin. **E** Representative photos of Lgr5 HFSCs regeneration in 3W Lgr5 + /mTmG mice. ****P < 0.001

Previous studies indicated that HFs are not able to regenerate if the wound diameter is less than 0.5 cm even in 3W mice [4]. To explore the effects of IL-1α in HFs de novo regeneration, a 0.5-cm-diameter wound was cut on the dorsal skin and then IL-1α was infused into the wound (Fig. 5A). As expected, PBS-treated mice did not regenerate HFs, whereas IL-1α induced HFs de novo regeneration (Fig. 5B, C). To confirm the results, tissue slides were stained by H&E. Results showed that the HFs already grew out from the original skin, and regenerated HFs were growing in the regenerated skin and a few regenerated HFs have grown out from the epidermis in the center of the regenerated skin (Fig. 5D). Moreover, the growth rate of HFs in the central region is faster than that in the peripheral region. Furthermore, Lgr5 + /mTmG mice showed similar results. There were almost no Lgr5 + HFSCs in the regenerated tissues of PBS-treated mice, but Lgr5 + HFSCs were present in the regenerated tissues of IL-1α-treated mice (Fig. 5E). It indicates that IL-1α can accelerate the de novo regeneration HFs and Lgr5 HFSCs.

### IL-1α induces HFs de novo regeneration through regulating inflammation and ROS productions in 3W mice

**Fig. 6:**
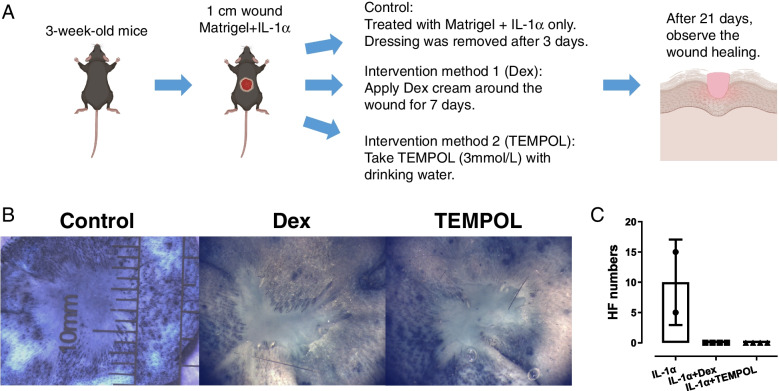
IL-1α induces de novo HFs regeneration by regulating inflammation and ROS productions. **A** Experimental steps. **B**, **C** Dexamethasone and TEMPOL inhibited the effects of IL-1α. Dex, dexamethasone

Although the above results indicate that IL-1α can promote de novo regeneration of HFs, however, it still does not explain the relationship between IL-1α and inflammation in the regenerative microenvironment. Previous studies have shown that IL-1α is a key cytokine that induces inflammation. In addition, ROS products generated by inflammation are also key to promote tissue regeneration. To investigate whether IL-1α promotes HF regeneration through inflammation and oxidative stress, we established a new research model. Simultaneous with IL-1α treatment, inflammatory and oxidative stress responses were inhibited with dexamethasone and TEMPOL, respectively (Fig. 6A). In addition, the morphology of the regenerated tissue varied considerably. The skin in the control group was closer in appearance to the original skin, whereas the Dex and TEMPOL treated groups differed considerably from the original skin in appearance. It showed that dexamethasone and TEMPOL reversed the effects of IL-1α (Fig. 6B, C). It suggests that IL-1α induces HFs de novo regeneration by regulating inflammation and oxidative stress.

### IL-1α activates HFSCs in 7W mice

De novo regeneration mainly involves dedifferentiation, transdifferentiation, and redifferentiation of skin cells. IL-1α accelerates this process and induces HFSCs de novo regeneration. However, HFSCs de novo regeneration does not mean that HFs will grow out. Generally, HFSCs in the traumatic margin area will migrate to the traumatic surface and reprogrammed into epidermal progenitors for epithelialization and epidermal renewal [22–25]. Therefore, HFSCs need to be continuously activated to ensure their proliferation and hair re-growth. The transition of the HFs cycle is the process of activation and proliferation of HFSCs. This can be used to mimic the activation and proliferation of HFSCs during skin regeneration.

**Fig. 7:**
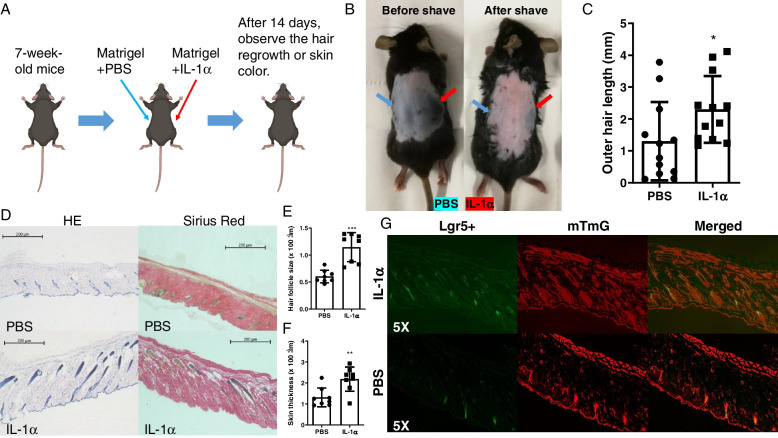
IL-1α stimulated hair follicle conversion from telogen to anagen phase and induced Lgr5 HFSCs proliferation in 7W mice. **A** Experimental steps. **B** Representative photos of mice on the 14th day after treatment. Blue arrows direct the control (PBS) area, and red arrows direct the treatment (IL-1α) area. **C** IL-1α increased outer hair length (PBS vs IL-1α, 1.304 ± 0.3546 vs 2.298 ± 0.3026, *n* = 12) (D) Representative pictures of HE and Sirius Red staining from the same mouse. **E** IL-1α increased HFs size. (PBS vs IL-1α, 0.6032 ± 0.04112 vs1.146 ± 0.09502, *n* = 8). **F** IL-1α increased skin thickness (PBS vs IL-1α, 1.316 ± 0.1599 vs 2.184 ± 0.2040, *n* = 8). **G** Representative pictures from Lgr5 + /mTmG mice (*n* = 8). *P < 0.05, **P < 0.01, ***P < 0.005

Although the above results confirmed that IL-1α can induce the de novo HFSCs regeneration, the effect of IL-1α on the proliferation of HFSCs is still unclear. It is well known that wound healing is a constantly changing process. Therefore, we employed a non-injury model to study the effect of IL-1α on HFSCs to avoid microenvironmental changes affecting our judgment of the results. For this, IL-1α was injected into one side of the dorsal skin in 7W Lgr5 + /mTmG mice, PBS was also injected into the other side as control (Fig. 7A). Interesting, after 14 days, IL-1α significantly promoted hair re-growth (or the skin color changes from pink to black), and also increased the HFs size and skin thickness (Fig. 6B–F). Fluorescence microscopy results found that IL-1α significantly increased the proliferation of Lgr5 HFSCs (Fig. 7G). This suggests that IL-1α not only induces the Lgr5 HFSCs de novo regeneration, but also activates Lgr5 HFSCs and promotes their proliferation. In other words, IL-1α plays an important role in both stages of HFSCs regeneration.

### IL-1α activates HFSCs via regulating inflammation and ROS

**Fig. 8:**
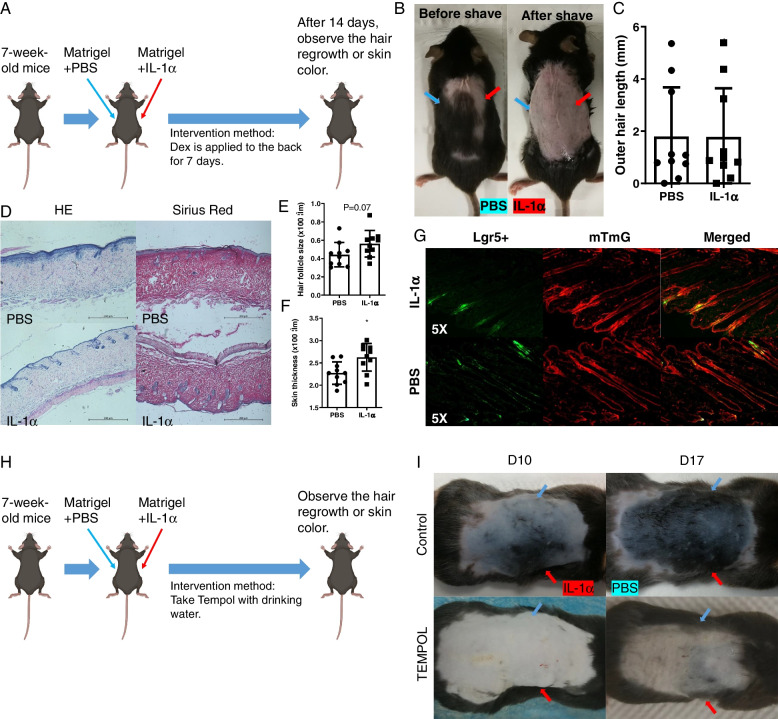
Dexamethasone and TEMPOL reduced IL-1α-induced effects in 7W mice. **A** Experimental steps. **B** Representative photos of mice on the 14th day after dexamethasone treatment. Blue arrows direct the PBS area, and red arrows direct the IL-1α area. **C** IL-1α failed to increase the outer hair length under the treatment of dexamethasone (PBS vs IL-1α, 1.796 ± 0.5952 vs 1.775 ± 0.5914, *n* = 10). **D** Representative pictures of HE and Sirius Red staining from the same mouse. **E** IL-1α did not increase HFs size (PBS vs IL-1α, 0.4420 ± 0.04205 vs 0.5618 ± 0.04586, *n* = 10). **F** IL-1α increased skin thickness (PBS vs IL-1α, 2.272 ± 0.07921 vs 2.625 ± 0.09812, *n* = 10). **G** Representative pictures from Lgr5 + /mTmG mice. **H** Experimental steps. **I** TEMPOL delayed IL-1α-induced hair regrowth. *P < 0.05, **P < 0.01

IL-1α is a proinflammatory cytokine, and inflammation regulates HFs regeneration [8, 35]. To identify whether IL-1α-induced HFs cycle transition and HFSCs proliferation via regulating inflammation, mice were also treated with dexamethasone (Fig. 8A). After treatment, there was no significant difference in hair length and HFs size between the two groups (Fig. 8B–E). However, IL-1α still increased the skin thickness (Fig. 8F). In Lgr5+/mTmG mice, IL-1α failed to activateLgr5 HFSCs proliferation under dexamethasone treatment (Fig. 8G). It indicates that IL-1α activates Lgr5 HFSCs by regulating inflammation, thereby promoting the regeneration of HFs.

Inflammation is often accompanied by oxidative stress, which in turn regulates tissue regeneration. To investigate whether IL-1α-activated the proliferation of HFSCs via regulating ROS productions, mice were also treated with TEMPOL (Fig. 8H). Generally, hair regrowth could be observed at day 10–14 post-IL-1α infusion. Compared to the control group, TEMPOL significantly delayed the hair regrowth time. While in TEMPOL group, IL-1α failed to activate hair regrowth (Fig. 8I). It suggests that IL-1α activates Lgr5 HFSCs by increasing ROS production, thereby promoting HFs regeneration.

### IL-1α promotes the proliferation of HaCaT and SKPs

**Fig. 9:**
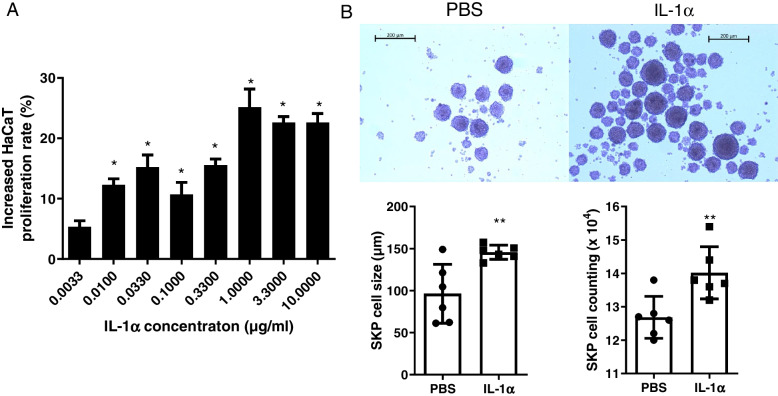
**A** IL-1α increased HaCaT cells proliferation (*n* = 6). **B** Representative pictures of SKP cells. IL-1α increased SKP cells aggregation and column numbers. **P < 0.01

HFs, as a skin appendage, cannot be regenerated without skin regeneration. Since IL-1α increased skin thickness (*P* < 0.05) and activated HFs regeneration (*P* = 0.07) even under dexamethasone treatment. We speculate that IL-1α may directly promote skin cells proliferation, which provides a good regenerative microenvironment for the regeneration of HFSCs. To further confirm our speculation, HaCaT and SKPs were treated with IL-1α. IL-1α significantly increased HaCaT cells proliferation (Fig. 9A), as well as increased SKPs aggregation and column numbers (Fig. 9B). Moreover, we also observed that IL-1α promoted the proliferation of HaCaT cells at low concentrations, but instead tended to inhibit cells proliferation when the concentration exceeded 1 μg/ml. This suggests that IL-1α can directly promote stem cells proliferation, which may provide the basis for regeneration of wounds tissue in the early stages and also help to promote the proliferation of HFSCs in the later stages.

## Discussion

The existence of HFs not only provides skin functional support, but also an important basis for maintaining skin homeostasis and self-renewal. Post-traumatic HFs reconstruction is very important to improve the quality of life of the patients. Based on the classic age-dependent regeneration model of HFs, this study analyzed the differences in the skin regeneration microenvironment and screened out the key exocrine protein IL-1α that affects the HFs regeneration. Furthermore, through various in vitro and in vivo investigations, we confirmed that IL-1α promotes de novo regeneration, activation, and proliferation of HFSCs by mediating inflammation and oxidative stress responses; and IL-1α can directly promote the proliferation of skin cell populations. We believe that IL-1α is a key factor to improve wound healing quality and HFSCs regeneration.

Wound healing is an extremely complex process [6]. The regeneration of each cell type is inseparable from the corresponding stem cell niche, which is usually not fixed but a constantly changing process. Here, we found similar results that the skin and HFs regenerative ability correlates with the regenerative microenvironment, especially inflammatory cells and associated signaling pathways. The role of inflammatory cells in tissue regeneration has been demonstrated by multiple studies. Typically, following tissue injury, monocytes migrate to the injury site where it is converted into pro-inflammatory macrophages (M1), which are responsible for cleaning the tissue and releasing a variety of cytokines [36]. Afterwards, the M1 phenotype decreases and the M2 phenotype gradually increases, accelerating tissue repair [37]. The M1/M2 ratio changes dynamically during post-injury repair. Stimulation of macrophages can release several cytokines, such as TNF-α, vascular endothelial growth factor, fibroblast growth factor, PDGF, TGF-β, and TGF-α [3, 38]. Dysfunction of macrophages may lead to abnormal repair, including disturbed secretion of inflammatory mediators and growth factors, and failure of communication between macrophages and surrounding cells, all of which lead to abnormal pathological fibrosis [39]. However, the phenotype of macrophages is different, and the release of biological factors is also different. Relevant studies are very limited [40]. In the present study, 3W mice had a higher M1/M2 ratio in the early stage of injury, and then the M1/M2 ratio gradually decreased, indicating that 3W mice could clear the injured tissue faster and have longer tissue repair time. This study also found that Tregs and TGF-β1 also changed dynamically after wounding, which further confirmed our belief. Moreover, in inflammatory responses, type 17 helper T cells-released IL-17 often acts in concert with IL-1, TNF-α, and inflammatory cells, and stimulates keratinocyte proliferation [41–43]. This may help to accelerate wound epithelialization and repair of the skin. Our findings also confirmed differences in the expression of IL-17, IL-1, and TNF-α. These dynamically changing interaction networks provide the basis for the high regenerative ability of the skin and its accessory organs in younger populations. Therefore, we believe that the involvement and dynamic changes of inflammatory cells contribute to the maintenance of a normal regenerative microenvironment. Age may affect inflammatory cell function and disrupt the regenerative microenvironment.

Previous studies reported that IL-1α promotes tissue and stem cell regeneration. For example, wound induces HFSCs regeneration and skin thickness through IL-1α-dependent activation of γδT cells [6]. Deletion of IL-1R reduces the effect of IL-1α. Moreover, T cells-secreted IL-1α promotes muscle stem cell proliferation in vivo after injury and stimulates the continuous expansion of muscle stem cells in vitro [44]. In a lacrimal gland injury model, IL-1α significantly increases cell proliferation, which are mainly inflammatory cells on days 1 and 2, and tissue cells on day 5 [45]. Compared to mechanical injury, IL-1α causes only temporary mild tissue injury and accelerated tissue regeneration. Furthermore, IL-1α promotes the regeneration of bone marrow mesenchymal stem cells by increasing IL-1 receptor 2 expression level [46]. Many studies have also confirmed that the activation of HFSCs is related to IL-1α, but no clear evidence has been presented [47–49]. Compared with these studies, this study not only confirmed that IL-1α can promote the HFSCs proliferation, but also promote the HFSCs de novo regeneration. However, we also found that IL-1α directly promoted the proliferation of HaCaTs and SKPs independent of inflammatory cells and oxidative stress. Due to the diversity of cell types in skin tissue, the sensitivity of different cells to IL-1α requires further research. Therefore, we believe that IL-1α can promote HFSCs regeneration and skin tissue proliferation.

Another important discovery is finding the mechanism of IL-1α in promoting HFSCs regeneration. The IL-1 family is closely related to the inflammatory response and proliferative stages [50]. Generally, IL-1α is particularly abundant in the cytokine profile of activated macrophages, neutrophils, epithelial cells, and endothelial cells. Keratinocytes, in particular, release large amounts of IL-1α following tissue injury or stress-related stimuli [51, 52]. The transcription factors AP1 and NF-κB can also induce IL-1α expression in a cell-type-specific manner [53–55]. Additionally, IL-1α plays a key role in the early inflammatory phase of the wound healing response, such as promoting macrophage proliferation and maturation [56, 57]. Also, IL-1α can directly promote the activation of CD(8) + T cells and natural killer cells [58]. This suggests that inflammation in the early stage of injury can promote the release of IL-1α, which can, in turn, activate inflammatory cells. In addition, IL-1α is important in mediating skin stem cell proliferation and stimulating the secretion of related growth factors, such as granulocyte–macrophage colony-stimulating factor and keratinocyte growth factor [59, 60]. The abundance of skin stem cells pools contributes to wound healing [61, 62]. Similarly, we found that IL-1α is highly expressed immediately after injury and persists for a long period of time. During this process, the dynamic changes of inflammatory cells were significantly different. Subsequent dexamethasone intervention experiments confirmed that IL-1α promoted the regeneration and proliferation of HFSCs by activating the inflammatory response. Inflammatory cells have been reported to be involved in the HFs cycle and the HFSCs proliferation. For example, macrophages promote Lgr5 HFSC and HF regeneration by inducing AKT/b-catenin signaling to release TNF-a after injury [35]. Skin-resident Tregs-expressed Jagged1 promotes HFSC proliferation and HFs regeneration [31]. Thus, we believe that IL-1α activates HFSCs by mediating inflammatory cells.

Furthermore, the present study found that oxidative stress is also important for IL-1α-induced HFs regeneration. Previous studies have shown that ROS signaling can regulate stem cell proliferation, differentiation, quiescence, apoptosis, and mobilization in various stem cell populations in a stage- and dose-dependent manner [63, 64]. Moreover, ROS can directly regulate the activation and apoptosis of HFSCs [65–71]. Here, TEMPOL inhibited IL-1α-induced HFs de novo regeneration, HFSCs activation, and hair growth by disturbing the balance of the stem cell niche. Conversely, some studies revealed that reducing oxidative stress is beneficial for wound healing and HFs regeneration [72, 73]. This may be due to different models. It is well known that aging increases the level of oxidative stress in the wound and thus disrupts the regenerative microenvironment [74]. Therefore, reducing oxidative stress is beneficial for wound healing [75], which provides a better tissue environment for HFSCs regeneration. However, complete suppression of oxidative stress is also not conducive to tissue regeneration and HFs regeneration [67]. Tissue regeneration can only be promoted by maintaining an appropriate oxidative stress environment. The higher enzymatic activity in the younger population can precisely regulate oxidative stress and create a microenvironment more conducive to tissue regeneration.

Interestingly, HFs regeneration was often observed in the center of the regenerated tissue, these results are in line with our findings [4]. Compared to the original skin, most of the regenerated HFs were still growing in the skin, and just a few HFs have grown out from the epidermis in the center of the regenerated tissue. This may be attributed to the fact that AP reagents can stain HFs that have grown from the epidermis, but cannot penetrate the epidermis to disseminate HFs, that are still growing in the skin. This is the reason why the results of AP staining results are not consistent with those of tissue sections. Similar results have been reported in a recent study. The HFs grew faster in the central part of the regenerated skin and grew slowly in the marginal part [7]. Unlike our research methods, they collected the samples on day 35 post-injury, while we collected samples on day 21 post-injury. Therefore, we believe that the regenerative microenvironment changes with distance from the wound margin. A regenerative microenvironment away from the wound edge is more likely to induce de novo regeneration of HFSCs.

This study has some limitations. We still do not know where the regenerated HFSCs originated from. There is still no evidence that IL-1α directly promotes the transdifferentiation, dedifferentiation, or differentiation of other cells into HFSCs. Therefore, we made three assumptions. First, IL-1α may promote the migration of HFSCs from wound margins. Second, IL-1α leads to regeneration and proliferation of HFSCs by regulating the regenerative microenvironment. Third, a small number of HFSCs were generated through dedifferentiation or transdifferentiation after injury, and IL-1α further expanded the proliferation of HFSCs. In either case, the proof is very difficult and further research is needed. Next, there is limited research on the effects of IL-1α on HFSCs at the cellular level, which is mainly due to the lack of a mature in vitro culture system for HFSCs. Additionally, the safe dose, pharmacokinetics, and pharmacometabolism of IL-1α require further investigation. Besides inflammation regulation and oxidative stress, more pharmacological mechanisms need to be explored.

## Conclusion

**Fig. 10:**
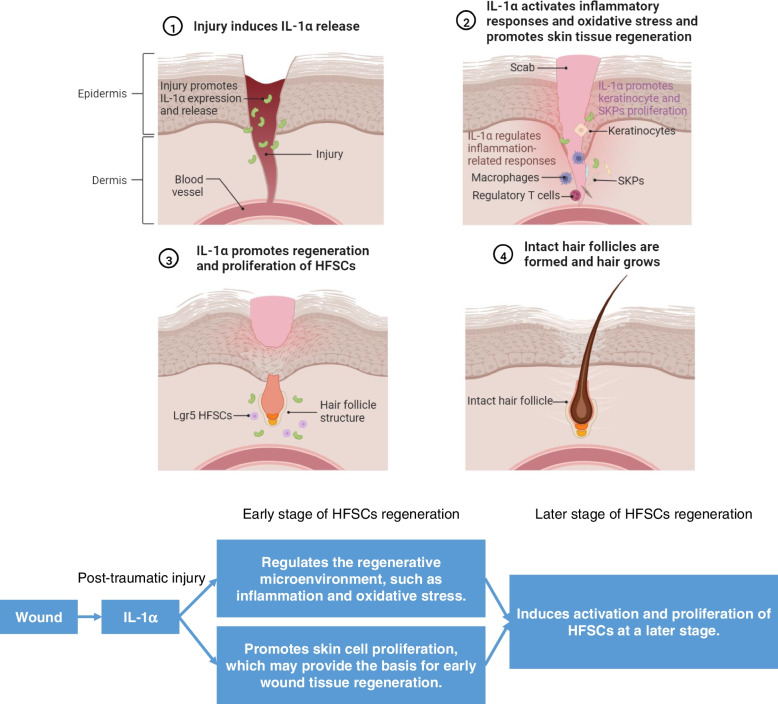
Schematic diagram of IL-1α promoting de novo hair follicle regeneration after injury

In conclusion, age alters the regenerative microenvironment by affecting inflammatory cells, oxidative stress, and cytokine levels, thereby reducing the regenerative capacity of skin and HFs. It suggests that IL-1α secreted in the early stage of injury regulates the regenerative microenvironment by driving inflammation and oxidative stress, and promotes the proliferation of skin cell populations to provide the basis for HF regeneration, thereby accelerating the de novo regeneration of Lgr5 HFSCs (Fig. 10). Furthermore, IL-1α could directly activate Lgr5 HFSCs and promote their proliferation, thereby promoting hair regrowth. IL-1α plays an important role in both regeneration and proliferation stages of HFs and is indispensable for the growth and maturation of HFs. These findings suggest that IL-1α is a promising protein drug in the treatment of alopecia, wound healing, and other degenerative skin diseases.

## Data Availability

The data used to support the finding of this study are available from the corresponding author upon request.

## Abbreviations

AP: Alkaline phosphatase
BSA: Bovine serum albumin
Dex: Dexamethasone
DP: Dermal papilla
FACS: Fluorescence-activated cell sorting
γδT-cells: Gamma delta T cells
GO: Gene Ontology
GFP: Green fluorescent protein
HF: Hair follicles
HFSC: Hair follicle stem cells
HaCaT: Human epidermal keratinocyte line
H&E: Hematoxylin and eosin
IL-1α: Interleukin-1alpha
IL-1β: Interleukin-1beta
IL-1R: Interleukin-1receptor
IL-17: Interleukin-17
KEGG: Kyoto Encyclopedia of Genes and Genomes
Lgr5: Leucine-rich repeat G protein-coupled receptor 5
PBS: Phosphate-buffered saline
PCR: Polymerase chain reaction
qRT-PCR: Quantitative reverse transcription PCR
ROS: Reactive oxygen species
SCDCP: Shenzhen Center for Disease Control and Prevention
SKPs: Skin-derived precursors
TNF-α: Tumor necrosis factor-alpha
Tgf-β1: Transforming growth factor-beta 1
Tregs: Regulatory T cells
